# Testing a Capacity-Load Model for Hypertension: Disentangling Early and Late Growth Effects on Childhood Blood Pressure in a Prospective Birth Cohort

**DOI:** 10.1371/journal.pone.0056078

**Published:** 2013-02-06

**Authors:** Carlos S. Grijalva-Eternod, Debbie A. Lawlor, Jonathan C. K. Wells

**Affiliations:** 1 UCL Institute for Global Health, London, United Kingdom; 2 MRC Centre for Causal Analysis in Translational Epidemiology, Department of Social Medicine, University of Bristol, Bristol, United Kingdom; 3 MRC Childhood Nutrition Research Centre, UCL Institute of Child Health, London, United Kingdom; Brigham and Women's Hospital and Harvard Medical School, United States of America

## Abstract

**Background:**

In 2005, it was estimated that hypertension affected 26.4% of the adult population worldwide. By 2025, it is predicted that it will affect about 60% of adults, a total of 1.56 billion. Both pre- and postnatal growth patterns have been associated with later blood pressure (BP), but in contrasting directions. These inconsistent associations of growth during different developmental periods merit elucidation. We tested a theoretical model treating birth weight as a marker of homeostatic metabolic capacity, and childhood height, lean mass and fat mass as independent indices of metabolic load. We predicted that decreased capacity and increased load would be independently associated with increased BP.

**Methods and Findings:**

Data from the ALSPAC cohort on growth from birth to 7 years, and body composition by dual-energy X-ray absorptiometry and BP at 9 years, were analysed (n = 6579). Data were expressed as standard deviation scores (SDS) or standardised regression residuals (SRR). BP was independently and positively associated with each of height, lean mass and fat mass. In a joint model systolic BP was positively associated with conditional weight velocity [males 0.40 (95%CI: 0.37–0.44) & females 0.44 (95%CI: 0.40–0.47) SDS/SRR], but not birth weight [0.00 (95%CI: −0.03–0.04) & 0.03 (95%CI: −0.01–0.07) SDS/SDS]. Adjusting for height, lean mass and fat mass, the association of systolic BP and conditional weight velocity attenuated [0.00(95%CI: −0.09–0.08) & −0.06(95%CI: −0.14–0.03) SDS/SRR], whereas that with birth weight became negative [−0.10 (95%CI: −0.14–0.06) & −0.09 (95%CI: −0.13–0.05) SDS/SDS]. Similar results were obtained for diastolic BP and pulse pressure.

**Conclusions:**

Consistent with our theoretical model, high metabolic load relative to metabolic capacity is associated with increased BP. Our data demonstrate the contribution of different growth and body composition components to BP variance, and clarify the developmental aetiology of hypertension.

## Introduction

Birth weight and growth in early life have been associated with later blood pressure (BP) in numerous studies, but the details of such associations remain controversial [1]. Initial studies linked low birth weight with greater BP later in life [2], but interpretation is complicated because such associations tend to emerge most strongly after adjustment for current body size [3]. A recent meta-analysis of 80 studies found that BP from childhood onwards fell with increasing birth weight, the size of the effect being approximately 2 mmHg per kg, but further showed that faster infant growth was positively associated with later BP [4]. Nutrition during infancy has also been associated with later BP, with both term and preterm infants randomised to formula milks with higher protein and energy content having higher BP in childhood or adolescence [5], [6].

Both birth weight and rapid infant growth have been associated with later obesity as measured by body mass index (BMI) [7], [8], [9], [10], although a recent meta-analysis showed no systematic association of low birth weight and later obesity [11]. Higher BMI is also associated with increased BP during childhood and adolescence [12]. However, BMI cannot differentiate the various components of nutritional status, including body frame size, lean mass (LM) and adiposity [13]. Previous studies show that growth in different developmental periods has variable associations with later BMI [14], [15], but childhood BMI conceals these specific growth effects, and their possible influence on BP.

Few studies have explored the detailed contribution of body composition to BP variance. One study showed that sex differences in adult BP disappeared when adjusted for LM [16]. Most studies investigating associations of body composition and BP have either not adjusted LM and fat mass (FM) appropriately for variance in body size or used inappropriate outcomes such as percentage fat, which is statistically problematic because FM is present in both numerator and denominator [17], [18].

How childhood BP is influenced by early growth patterns and the accretion of different tissue masses therefore remains unclear. This situation may be improved by incorporating a theoretical perspective attempting to clarify how growth during different developmental periods affects physiological parameters that regulate and challenge BP.

The thrifty phenotype hypothesis of Hales and Barker proposed that the small baby adapts to poor nutritional supply in-utero by reducing investment in vital organs such as the pancreas and kidney [19]. This reduced investment then reduces toleration of higher levels of homeostatic load, manifesting as increased body weight later in life. Whereas the thrifty phenotype model emphasised differences in growth between individuals of low and normal birth weight, we have developed a model emphasising continuous associations between body size, in different periods of the life course, and physiological parameters. Specifically, we have proposed a model for chronic disease risk emphasising birth weight as an index of ‘metabolic capacity’ (components of organ physiology promoting the maintenance of homeostasis), and several factors in childhood (large tissue masses, sedentary behaviour and lipogenic diet) as indices of ‘metabolic load’ that challenge the ability to maintain homeostasis [20], [21]. According to this model, greater body size and adiposity are predicted to increase metabolic load, whereas low birth weight is predicted to reduce metabolic capacity, for example through reductions in nephron number which scales linearly with birth weight [22]. In our study we also treat height as a marker of load, because greater body size imposes greater functional load on organs, in this case, kidneys [23]. A high load relative to a given capacity is then predicted to increase BP.

**Figure 1:**
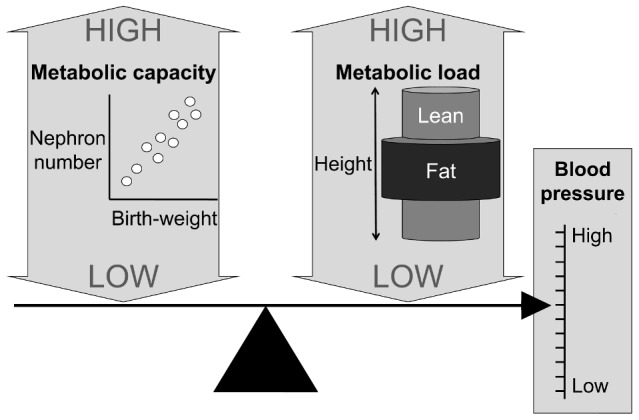
Diagram illustrating the concepts of metabolic capacity and metabolic load in relation to blood pressure. Metabolic capacity is indexed by nephron number, which scales positively with birth weight. Metabolic load is indexed by each of height, lean mass and fat mass, which are portrayed using a cylinder model, in which cylinder lengths are proportional to height, and the volumes are proportional to the masses of lean and fat mass. A high load and a low capacity are each predicted to increase blood pressure.

Building on previous work in adults that demonstrated independent associations of birth weight and adult height or BMI, with adult BP [24], we tested our capacity-load model by disentangling the contributions of pre- and postnatal growth and childhood body composition to childhood BP. We tested the hypothesis that three different components of metabolic load (height, relative LM and relative FM) are independently positively associated with childhood BP. We further tested the hypothesis that postnatal growth is positively associated with childhood BP via increased metabolic load, whereas independent of that effect, birth weight, as a marker of metabolic capacity, is inversely associated with BP. This conceptual framework is illustrated in **Figure 1**.

## Methods

### Study population

We used data from the Avon Longitudinal Study of Parents and Children (ALSPAC). A full description of the methods is reported elsewhere [25], [26], see also www.bristol.ac.uk/alspac. Briefly, ALSPAC is a prospective cohort study, investigating the health and development of children, which enrolled 14,541 pregnant women residing in three districts in Bristol, England, with expected delivery dates from 1 April 1991 to 31 December 1992. Data was obtained using self-administered questionnaires, data extraction from medical notes, linkage to routine information systems and through research clinics attendance.

### Ethics statement

Ethical approval for the study was obtained from the ALSPAC Ethics and Law Committee and the Local Research Ethics Committees; written consent was obtained from all participants and their parents/guardians.

### Data available

The ALSPAC study included regular assessments from 7–15 years for the whole cohort, although the nature of data collected changed over time. In the current analysis we examined data from 6,621 singletons (3,285 males) with complete data obtained at 9 years for sex, weight, height, body composition and BP. For this group, weight and length/height data were also available at birth (n = 6,546 and 5,272, respectively) and at 7 years (n = 5,826 and 5,830, respectively).

Anthropometric data were available from several sources. Birth weight was extracted from medical records. Birth length (crown-heel) was measured by ALSPAC staff who visited newborns soon after birth (median 1 day, range 1–14 days), using Harpenden Neonatometers (Holtain Ltd, Crymych, UK). Gestational age was obtained from self-reported date of last menstrual period. At each further assessment, weight and height were measured with the child in light clothing, without shoes. Weight was measured to the nearest 0.1 kg using Tanita scales (Tanita UK Ltd, Middlesex, UK) and height to the nearest 1 mm using Harpenden stadiometers (Holtain Ltd). LM and FM were assessed by dual-energy X-ray absorptiometry (Lunar Prodigy, GE Medical Systems, Madison, WI, USA). BP was measured with Dinamap 9301 Vital Signs Monitors (Morton Medical, Cirencester, UK) with correct cuff sizes. Two readings of systolic and diastolic BP were recorded, with the child at rest with arm supported, and the mean of each calculated.

### Conceptual approach to body composition

Previous work has shown that each of height, LM and FM may be positively associated with BP [16], [27], [28]. To disentangle their relative contributions, we adjusted LM relative to height, and FM relative to LM, on the following conceptual basis as illustrated in **Figure 1**. Treating the body as cylindrical in form, we first addressed cylinder length, i.e. height. We then considered variability in LM relative to height, i.e. the volume of LM relative to height. Thus, for a given height, we assume greater BP is required to distribute blood to a larger LM. Finally, as LM is the primary determinant of energy requirements [29], [30], we assumed that the amount of energy required to withstand starvation for a given unit of time scales positively with LM, as demonstrated by a tight association between LM and FM across mammal species [31]. We therefore adjusted FM relative to LM or relative to height, on the assumption that for a given LM, or height, a higher FM provides more energy, but at a cost of greater metabolic load.

### Data handling

Previous evidence has shown that height is positively associated with both LM and FM, which in turn are positively associated [18]. The strength of these associations differs by sex. To remove the effect of these associations from our analysis, we adopted a modified version of a method known as unexplained residuals to generate independent outcomes [32]. Briefly, by sex, LM was regressed against height. Outlier detection was undertaken using Hadi's method [33], participants with outlying values were removed from further analyses (two outliers). Predicted LM was then obtained using the regression output values, and LM standardised residuals modelled on height (LMr/H) were calculated as the difference between observed and predicted LM (unexplained residuals), divided by the single standard deviation of the unexplained residuals distribution. A similar approach was used to obtain standardised residuals for weight regressed against height (Wr/H, one outlier), and FM regressed against height (FMr/H, one outlier), and against LM (FMr/LM, 23 outliers).

Anthropometric data at birth, 7 and 9 years were converted to UK 1990 z-scores [34]. Growth was expressed as conditional weight or height velocity (CWV and CHV, respectively), using the same standardised residuals approach, adjusting 7-year data for equivalent data on birth weight or length respectively. We removed three outliers for CWV and nine for CHV. Pulse pressure (PP), considered to be an important and independent predictor of cardiovascular disease risk [35], was calculated as the difference between systolic and diastolic BP. PP, and systolic and diastolic BP, were also converted to z-scores, based on BP centiles for UK children [36]. All subjects identified as outliers were excluded from our analysis (n = 42).

The above approach converted all data into a comparable format, equivalent to z-scores, making it easier to compare information across different traits with different magnitudes of variance. To simplify for the reader and aid interpretation, the terms ‘standardised residual’ and ‘z-score’ were used to differentiate between those derived based on our sample, and those based on published normative data, respectively.

### Statistical analysis

For each sex, we then used regression analysis to evaluate the contribution of the various body components (current metabolic load: height z-score; Wr/H, LMr/H, FMr/H and FMr/LM) to variance in BP outcomes. We then evaluated the independent contributions of prenatal growth (birth weight or length z-score, considered as metabolic capacity) and postnatal growth (CWV or CHV, considered as a proxy for metabolic load in early life). Finally, pre- and postnatal growth data for either weight or length/height were added to the model including the components of current metabolic load.

To understand the possible contributions of metabolic capacity and load to children considered to have higher BP, placing them at a higher risk of future cardiovascular disease [37], [38], we specified two groups according to the cut-off criteria of the Working Group on High Blood Pressure in Children and Adolescents [39], as normal and high (<95th and ≥95th centile, respectively) for systolic and diastolic derived BP outcomes. We then evaluated the odds ratios of different components of metabolic capacity and load for higher BP using logistic regression. As hypertension is defined clinically on the basis of three BP readings on three different occasions, and our protocol measured BP on a single occasion, and might be influenced by ‘white coat effect’, our definition of ‘higher BP’ is not equivalent to hypertension as defined by the Working Group, even though it was categorised using the same cut-off, and simply categorises a group with higher BP within our sample. All analyses were undertaken using Stata (StataCorp. 2007. Stata Statistical Software: Release 10. College Station, TX: StataCorp LP).

## Results

Characteristics of interest of the sample taken at birth, 7 years, and 9 years are described in **Table 1**.

**Table 1:** Subject characteristics by sex.

	Males			Females		
	n	mean	s.d.	n	mean	s.d.
Gestational age at birth (weeks)	3261	39.4	1.8	3318	39.6	1.7
Birth weight (kg)	3225	3.49	0.55	3280	3.39	0.49
Length at birth (cm)	2599	51.1	2.4	2639	50.4	2.2
Birth weight (z-score)	3225	0.11	1.00	3280	0.12	0.97
Length at birth (z-score)	2589	0.23	1.06	2630	0.22	1.11
Age at 7 years (months)	2908	89.7	2.1	2923	89.7	2.1
Weight at 7 years (kg)	2889	25.5	4.1	2897	25.6	4.4
Height at 7 years (cm)	2892	126.0	5.2	2898	125.1	5.3
Weight at 7 years (z-score)	2889	0.21	1.02	2897	0.17	0.99
Height at 7 years (z-score)	2892	0.25	0.96	2898	0.18	0.98
Age at 9 years (months)	3261	118.5	3.9	3318	118.4	3.9
Weight at 9 years (kg)	3261	34.3	6.8	3318	34.9	7.5
Height at 9 years (cm)	3261	140	6	3318	139	6
Weight at 9 years (z-score)	3261	0.39	1.02	3318	0.31	1.04
Height at 9 years (z-score)	3261	0.35	0.96	3318	0.25	0.98
Lean mass at 9 years (kg)	3261	25.5	2.9	3318	23.6	3.1
Fat mass at 9 years (kg)	3261	7.3	4.6	3318	9.6	4.9
Systolic blood pressure (mmHg)	3261	102.5	9.0	3318	102.9	9.6
Diastolic blood pressure (mmHg)	3261	57.2	6.4	3318	57.7	6.4
Pulse pressure (mmHg)	3261	45.3	7.6	3318	45.2	7.9
Systolic blood pressure (z-score)	3261	−0.70	1.01	3318	−0.75	1.04
Diastolic blood pressure (z-score)	3261	0.09	0.77	3318	0.08	0.79
Pulse pressure (z-score)	3261	−0.76	0.85	3318	−0.84	0.88

### Current metabolic load and BP

The contributions of the different body components to BP, by sex, are shown in **Table 2**. In males, height z-score showed a positive association with diastolic BP z-score (Model 1). Inclusion of Wr/H to the model (Model 2) did not substantively alter this association, with Wr/H showing an independent positive association with diastolic BP z-score. Including LMr/H and FMr/LM instead of Wr/H (Model 3) did not alter the explanatory power of the model; however, while FMr/LM independently contributed positively to diastolic BP z-score variance, LMr/H did not. A similar pattern was observed in females, except for LMr/H in Model 3 which showed a small but positive association with BP (although there was no strong statistical evidence of a sex*LMr/H interaction; p = 0.49).

**Table 2:** Linear regression models for the prediction of blood pressure outcomes by height, weight, lean and fat mass.

	DIASTOLIC BLOOD PRESSURE (z-score)											
	*Males (n = 3,261)*						*Females (n = 3,318)*					
	Model 1		Model 2		Model 3		Model 1		Model 2		Model 3	
	β	95% CI	β	95% CI	β	95% CI	β	95% CI	β	95% CI	β	95% CI
Height*	0.12	(0.09; 0.15)	0.12	(0.10; 0.15)	0.11	(0.09; 0.14)	0.11	(0.08; 0.13)	0.11	(0.08; 0.14)	0.11	(0.08; 0.14)
Wr/H			0.12	(0.10; 0.15)					0.15	(0.12; 0.18)		
LMr/H					0.02	(−0.01; 0.04)					0.03	(0.00; 0.06)
FMr/LM					0.15	(0.12; 0.17)					0.17	(0.14; 0.19)
*r^2^*	0.02		0.05		0.06		0.02		0.05		0.06	

	SYSTOLIC BLOOD PRESSURE (z-score)											
	*Males (n = 3,261)*						*Females (n = 3,318)*					
	Model 1		Model 2		Model 3		Model 1		Model 2		Model 3	
	β	95% CI	β	95% CI	β	95% CI	β	95% CI	β	95% CI	β	95% CI
Height*	0.30	(0.26; 0.33)	0.30	(0.27; 0.33)	0.29	(0.25; 0.32)	0.31	(0.27; 0.34)	0.31	(0.28; 0.34)	0.32	(0.28; 0.35)
Wr/H			0.33	(0.30; 0.36)					0.37	(0.34; 0.40)		
LMr/H					0.20	(0.17; 0.23)					0.23	(0.20; 0.26)
FMr/LM					0.28	(0.25; 0.31)					0.28	(0.25; 0.31)
*r^2^*	0.08		0.19		0.19		0.08		0.21		0.20	

	PULSE PRESSURE (z-score)											
	*Males (n = 3,261)*						*Females (n = 3,318)*					
	Model 1		Model 2		Model 3		Model 1		Model 2		Model 3	
	β	95% CI	β	95% CI	β	95% CI	β	95% CI	β	95% CI	β	95% CI
Height*	0.19	(0.16; 0.22)	0.19	(0.16; 0.22)	0.18	(0.15; 0.21)	0.22	(0.19; 0.25)	0.22	(0.19; 0.25)	0.22	(0.20; 0.25)
Wr/H			0.22	(0.19; 0.25)					0.25	(0.22; 0.28)		
LMr/H					0.18	(0.16; 0.21)					0.21	(0.18; 0.24)
FMr/LM					0.15	(0.12; 0.18)					0.15	(0.12; 0.17)
*r^2^*	0.04		0.11		0.11		0.06		0.14		0.14	

β: mean difference in outcome per 1 unit exposure, 95% CI: 95% confidence intervals, Wr/H: Weight standardised residuals modelled on height, LMr/H: Lean mass standardised residuals modelled on height, FMr/LM: Fat mass standardised residuals modelled on lean mass.

* Height is expressed in z-scores.

A similar pattern was observed for systolic BP z-score, but with explanatory variables showing larger regression coefficients and explaining more of the model variance. In males, height z-score showed a positive association and explained ∼8% of systolic BP z-score variance (Model 1). Inclusion of Wr/H (Model 2) increased the explanatory power of the model to ∼19%. When LMr/H and FMr/LM were included in the model instead of Wr/H (Model 3) all three components of load independently contributed positively to systolic BP z-score variance, together explaining a similar amount of variance (∼19%) as in Model 2. Similar findings were observed for females.

Likewise, in males and females, height z-score alone (Model 1) or height and Wr/H together (Model 2) independently contributed positively to PP z-score variance. Inclusion of LMr/H and FMr/LM in the model instead of Wr/H (Model 3) did not alter the explanatory power of the model, with all three components independently showing a positive association with PP z-score.

### Early growth and current metabolic load

**Figure 2:**
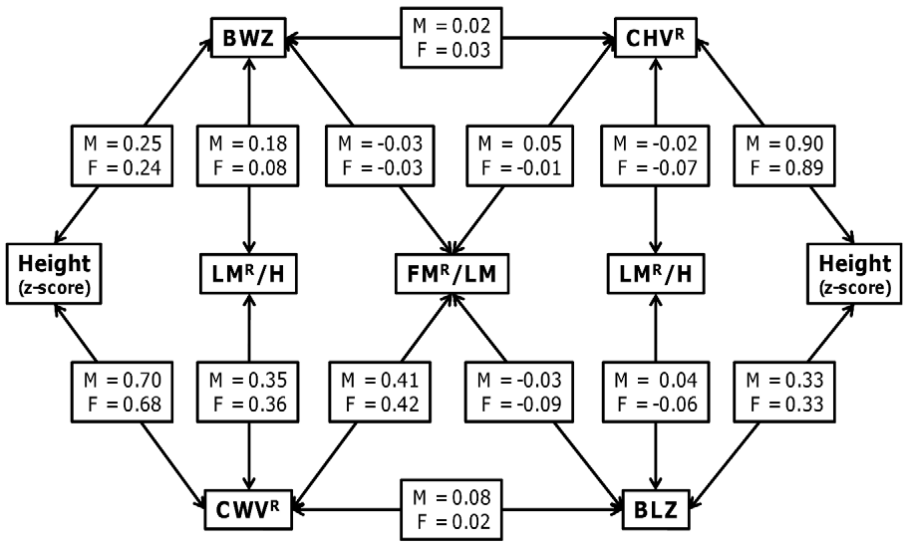
Correlations between pre- and postnatal growth indices and body composition at 9 years, for each sex (M = males; F = females). BLZ: Length at birth z-score; BWZ: Birth weight z-score; CHV Conditional height velocity; CWV: Conditional weight velocity; FMr/LM: Fat mass standardised residuals modelled on lean mass; LMr/H: Lean mass standardised residuals modelled on height.

**Figure 2** shows the correlations between pre- and postnatal growth indices and different body components at 9 years. Birth weight z-score showed a positive correlation with height z-score and LMr/H in both sexes, but no correlation with FMr/LM. CWV was positively correlated with all body components in both sexes, in each case showing stronger correlations than birth weight. Birth length z-score showed a strong correlation with height z-score in both sexes, but little correlation with LMr/H and FMr/LM. Likewise CHV showed a strong correlation with height z-score in both sexes, but little correlation with LMr/H and FMr/LM.

### Early growth, current metabolic load and BP

The contributions of pre- and postnatal growth in weight to BP, alone and in conjunction with the different body components are presented in **Table 3** for each sex. Model 1 evaluates current metabolic load alone, differentiating the contributions of height, LMr/H and FMr/LM (this is equivalent to Model 3 in Table 2). Model 2 evaluates the contribution of pre- and postnatal growth alone, i.e. metabolic capacity and early metabolic load, respectively. Model 3 includes both current metabolic load and pre- and postnatal growth. The sample size included in Table 3 is smaller than in Table 2 due to some missing data on anthropometric measurements at birth or at age 7 years. However, the results for Model 1 in Table 3 do not differ substantively from their equivalent results conducted using the larger sample (Model 3 in Table 2), suggesting no selection bias was introduced by using this smaller sample.

**Table 3:** Linear regression models for the prediction of blood pressure outcomes by height, weight, lean and fat mass, birth weight and conditional weight velocity.

	DISTOLIC BLOOD PRESSURE (z-score)											
	*Males (n = 2,855)*						*Females (n = 2,864)*					
	Model 1		Model 2		Model 3		Model 1		Model 2		Model 3	
	β	95% CI	β	95% CI	β	95% CI	β	95% CI	β	95% CI	β	95% CI
Height*	0.12	( 0.09; 0.15)			0.12	( 0.06; 0.19)	0.12	( 0.09; 0.15)			0.15	( 0.09; 0.22)
LMr/H	0.01	(−0.01; 0.04)			0.02	(−0.02; 0.06)	0.03	( 0.00; 0.06)			0.04	( 0.00; 0.08)
FMr/LM	0.14	( 0.11; 0.17)			0.13	( 0.09; 0.18)	0.16	( 0.13; 0.19)			0.18	( 0.14; 0.22)
Birth weight*			−0.01	(−0.04; 0.02)	−0.04	(−0.07; 0.00)			0.00	(−0.03; 0.03)	−0.04	(−0.08; −0.01)
CWV			0.15	( 0.12; 0.18)	0.01	(−0.06; 0.08)			0.16	( 0.13; 0.19)	−0.03	(−0.10; 0.04)
*r^2^*	0.05		0.04		0.05		0.06		0.04		0.06	

	SYSTOLIC BLOOD PRESSURE (z-score)											
	*Males (n = 2,855)*						*Females (n = 2,864)*					
	Model 1		Model 2		Model 3		Model 1		Model 2		Model 3	
	β	95% CI	β	95% CI	β	95% CI	β	95% CI	β	95% CI	β	95% CI
Height*	0.29	( 0.26; 0.33)			0.32	( 0.25; 0.40)	0.33	( 0.30; 0.37)			0.39	( 0.32; 0.47)
LMr/H	0.19	( 0.16; 0.22)			0.21	( 0.16; 0.26)	0.23	( 0.20; 0.27)			0.26	( 0.21; 0.31)
FMr/LM	0.26	( 0.23; 0.30)			0.26	( 0.21; 0.31)	0.29	( 0.25; 0.32)			0.31	( 0.26; 0.36)
Birth weight*			0.00	(−0.03; 0.04)	−0.10	(−0.14; −0.06)			0.03	(−0.01; 0.07)	−0.09	(−0.13; −0.05)
CWV			0.40	( 0.37; 0.44)	0.00	(−0.09; 0.08)			0.44	( 0.40; 0.47)	−0.06	(−0.14; 0.03)
*r^2^*	0.18		0.15		0.19		0.21		0.17		0.22	

	PULSE PRESSURE (z-score)											
	*Males (n = 2,855)*						*Females (n = 2,864)*					
	Model 1		Model 2		Model 3		Model 1		Model 2		Model 3	
	β	95% CI	β	95% CI	β	95% CI	β	95% CI	β	95% CI	β	95% CI
Height*	0.19	( 0.15; 0.22)			0.22	( 0.15; 0.29)	0.23	( 0.20; 0.26)			0.28	( 0.21; 0.34)
LMr/H	0.18	( 0.15; 0.21)			0.20	( 0.15; 0.25)	0.21	( 0.18; 0.25)			0.24	( 0.19; 0.28)
FMr/LM	0.14	( 0.11; 0.17)			0.15	( 0.10; 0.19)	0.15	( 0.12; 0.18)			0.17	( 0.12; 0.21)
Birth weight*			0.01	(−0.02; 0.04)	−0.07	(−0.11; −0.03)			0.03	( 0.00; 0.06)	−0.06	(−0.10; −0.02)
CWV			0.26	( 0.23; 0.29)	−0.02	(−0.10; 0.06)			0.30	( 0.27; 0.33)	−0.04	(−0.12; 0.03)
*r^2^*	0.11		0.09		0.11		0.15		0.11		0.15	

β: mean difference in outcome per 1 unit exposure, 95% CI: 95% confidence intervals, LMr/H: Lean mass standardised residuals modelled on height; FMr/LM: Fat mass standardised residuals modelled on lean mass; CWV: Conditional weight velocity.

* Birth weight and height are expressed in z-scores.

In Model 2, for all BP components in both sexes, CWV showed positive associations, whereas birth weight z-score did not show an association. Early growth explained ∼4% of diastolic BP z-score variance in both sexes, ∼15 and ∼17% of the variance of systolic BP z-score for males and females, respectively, and ∼9 and ∼11% of the variance of PP z-score for males and females, respectively. In Model 3, a similar pattern was observed for all BP components. The association of CWV with any component of BP was attenuated to the null with inclusion of current measures of metabolic load in both sexes, whereas an inverse association for birth weight z-score appeared. A similar pattern of results was observed when using length at birth z-score and conditional height velocity (**Table S1**).

Similar results as in Tables 3 and 4 were observed if FMr/H replaced FMr/LM in the models (results available from authors on request). In addition, when the sexes were jointly analysed, sex failed to present a significant contribution to any of the three BP components in all models (data not shown).

**Table 4:** Logistic regression for the odds of higher blood pressure.

DIASTOLIC BLOOD PRESSURE (z-score). *Values ≥95^th^ centile = 139*						
	Model 1		Model 2		Model 3	
	OR	95% CI	OR	95% CI	OR	95% CI
Height*	1.08	(0.91; 1.28)			1.08	(0.76; 1.53)
LMr/H	1.13	(0.96; 1.33)			1.12	(0.89; 1.41)
FMr/LM	1.57	(1.35; 1.82)			1.51	(1.21; 1.89)
Birth weight*			0.83	(0.70; 0.99)	0.80	(0.66; 0.98)
CWV			1.52	(1.29; 1.80)	1.08	(0.72; 1.63)
Male sex	0.88	(0.63; 1.24)	0.88	(0.63; 1.24)	0.88	(0.62; 1.24)

SYSTOLIC BLOOD PRESSURE (z-score). *Values ≥95^th^ centile = 94*						
	Model 1		Model 2		Model 3	
	OR	95% CI	OR	95% CI	OR	95% CI
Height*	1.68	(1.36; 2.08)			2.47	(1.66; 3.65)
LMr/H	1.78	(1.48; 2.14)			2.17	(1.69; 2.80)
FMr/LM	1.74	(1.49; 2.04)			2.09	(1.63; 2.68)
Birth weight*			1.01	(0.82; 1.24)	0.68	(0.53; 0.87)
CWV			2.67	(2.17; 3.28)	0.61	(0.37; 1.00)
Male sex	1.09	(0.72; 1.66)	1.13	(0.75; 1.71)	1.07	(0.70; 1.64)

OR: Odds ratio, 95% CI: 95% confidence intervals, LMr/H: Lean mass standardised residuals modelled on height; FMr/LM: Fat mass standardised residuals modelled on lean mass; CWV: Conditional weight velocity. Higher blood pressure (BP) for each component was defined using the cut-off criteria of ≥95^th^ centile, as recommended by the Working Group on High Blood Pressure in Children and Adolescents. However, clinical diagnosis of hypertension uses a different measurement protocol to our epidemiological approach, hence although derived using the same cut-off values, our higher BP sample does not represent a clinical hypertension sample. n = 5,719.

* Birth weight and height are expressed in z-scores.

### Metabolic capacity and load and higher BP

**Table 4** reports the odds for higher BP in children at 9 years, using a similar set of models as in **Table 3**. For diastolic BP, of the current components of metabolic load (Model 1) only an increase in FMr/LM increased the odds for higher BP. When evaluating pre- and postnatal growth in weight alone (Model 2) an increase of CVW or birth weight z-score increased and decreased the odds for higher BP, respectively. After inclusion of the current metabolic load components (Model 3) the odds for higher BP observed for CWV were attenuated to the null, while an increase in birth weight z-score continued to decrease the odds of higher BP.

For systolic BP an increase of all three current components of metabolic load increased the odds for higher BP (Model 1). For Model 2, an increase in CWV increased the odds of higher BP while birth weight z-score did not alter the risk. In Model 3 an increase in all three current components of metabolic load increased the odds for higher BP while an increase in birth weight z-score reduced the odds. In Model 3, the odds for higher BP for an increase of CWV were attenuated to the null.

When including birth length z-score and CHV in the modelling, similar patterns of results were observed (**Table S2**). The only difference was that birth length z-score did not alter the odds for higher BP in any model in which it was included.

## Discussion

In this study we have shown two important findings. First, all three components of childhood metabolic load, i.e. height, relative LM and relative adiposity, are independently associated with greater BP. In addition, all three components are associated with greater odds of higher BP as indexed by systolic BP, while only relative adiposity was associated with odds of higher diastolic BP. Second, when jointly evaluating the independent contribution of pre- and postnatal growth (as indexed by birth size and conditional growth velocity) to BP variance or the odds for higher BP, only postnatal growth contributed; however, after holding current metabolic load constant, the contribution of postnatal growth attenuated and an inverse association of birth weight became apparent. This indicates first that for a given metabolic load, a diminished metabolic capacity increases BP, and second that the association of postnatal growth on BP may be attributable to increased metabolic load.

Previously, most emphasis has been directed to the positive association of BMI or FM with BP [27], [40], [41] and few studies have expanded this approach to include LM as a contributing factor. One study of adults showed that adjusting for LM, the sex difference in BP disappeared [16], a finding we reproduced here. Positive associations between each of height, LM and FM with BP have been previously reported in children [28], [40], [42]. In the same cohort, for example, Brion et al. reported independent associations between BP and absolute values of LM and FM [42]. However, the strong colinearity between LM and FM, evident in our own analyses, has not previously been addressed. Lawlor and co-workers reported an increase in the odds of hypertension at 15–16 years with an increase of FM at 9–12 years [OR: 1.26 (95%CI: 1.14–1.40) for systolic BP per kg increase of FM] [40]. In our analysis we built on these findings by assessing how each component of metabolic load, independently and relative to each other, contributed to BP variance and the odds of higher BP, and how these associations interacted with growth patterns in early life.

The association between early growth patterns and later BP is complex, and improved conceptual models may help resolve seemingly contradictory findings. The known negative association between birth weight and BP is considered by some the most consistent evidence supporting the thrifty phenotype hypothesis [1]. However, concern has been expressed over statistical adjustments for current body size when quantifying the association of birth weight with later BP [3]. This problem was resolved by showing, in a large sample of Swedish men aged ∼18 years of similar BMI value, an inverse association between birth weight and systolic BP [43], confirming that early growth trajectories contribute significantly to later BP variance.

The association between adult height and cardiovascular disease risk remains uncertain, with both tall and short individuals proposed to have greater risk than those of intermediate height [44]. However, variance in the BP of tall adults is partly explained by variance in fetal growth. A study of Swedish men showed that in those of high birth weight, BP was lower in taller individuals; whereas in those of low birth weight, BP was lower in short individuals [24]. This Swedish study confirms that the effect of load (height) on BP varies according to metabolic capacity (birth weight). Our own study provides similar findings, and builds on this earlier work by showing that in the context of BP, each of height, LM, and adiposity represent components of metabolic load.

The concepts of metabolic capacity and load help to understand how growth during different periods influences BP. In relation to metabolic load, the associations of height, LM and FM with BP may derive from several physiological mechanisms. FM may contribute to BP either through hormonal mechanisms, such as perturbations of the renin-angiotensin-aldosterone system or leptin regulation [45], or though perturbations of the sympatho-renal axis [46]. For height and LM, allometric mismatch may be the potential mechanism. Weder and Schork reported that kidneys and blood volume scale with body size with an allometric constant of ≈0.8 and ≈1.0, respectively [23]. Therefore as the body grows, absolute kidney size lags behind blood volume, thus imposing a greater load on the kidneys. As a result the pressure-natriuresis ‘set point’, where sodium and water intake and output are balanced [47], rises as a mechanism of normalising plasma volume [48] and maintain renal homeostasis [23].

The physiological component of metabolic capacity most relevant to BP is likely to be nephron number, which has a tight positive association with birth weight over the majority of the birth weight range [22]. A reduced nephron number has been associated with increased BP [49], [50]. The burden of adiposity and allometric mismatch is thus predicted to be greater in those with reduced fetal growth [21], and our findings fit with this model.

In addition to our main analyses, we further investigated the odds ratio of components of metabolic capacity and load for higher BP. Whilst higher BP in children may derive from white coat effect as well as somatic properties, our data indicate that smaller birth size and larger size in childhood both contribute to the likelihood of having higher BP. Our results indicate a modest protective effect of birth weight, but substantial adverse effects of all three components of metabolic load. Thus, in a group characterised by higher BP in childhood, who are at increased risk of high BP in adulthood [37], [38], the primary childhood body composition phenotypes are large body size and adiposity.

The comparable contributions of metabolic load and postnatal growth to BP were different for diastolic compared to systolic BP or PP. First, metabolic load components showed greater regression coefficients and odds of higher BP for systolic BP and PP than for diastolic BP in both sexes. Second, the association between LM or height and diastolic BP was not consistent between the sexes, in contrast to systolic BP and PP. Our results indicate the possibility of independent pathways by which the different body components and postnatal growth impact on diastolic BP and PP.

The strengths of this analysis include the well characterised prospective cohort, the large sample size, the objective measures of body composition, and the use of the conceptual model of capacity and load. The limitations include some loss to follow-up, although this is unlikely to have adversely influenced our findings, limitations in the accuracy of DXA in association with fatness [51], and the fact that the postnatal growth rate was calculated over 7 years rather than specifically during infancy, though previous work in this cohort shows that growth in infancy is not a specific predictor for later BP or other cardiovascular disease risk factors [12]; a finding that has been replicated by others [52]. The ALSPAC cohort is also composed primarily of white European children, hence our findings may not necessarily generalise to other ethnic groups. Also, our use of a novel conceptual and statistical approach to characterise the independent contribution of each of height, LM and FM to BP may have introduced some bias in the results. For instance, derived standardised residuals did not remove the observed heteroskedasticity in the relation between FM and height or LM. However, our results remained unchanged after removing this heteroskedasticity from the models by log-transforming the data prior to calculating standardised residuals (data not shown). In addition, as we used several models, some statistically significant findings might be expected to emerge by chance. We believe this is not the case, as our findings changed very little across the different models (i.e. there is consistency); rather we used these models to help understand the patterns of associations between growth, body composition and BP variables.

Our findings are relevant to public health policies aimed at reducing the childhood antecedents of adult hypertension. Others have argued that rapid weight gain at any point in childhood is associated with greater BP [52]. However, different periods of growth may be relevant to different components of metabolic load. The fat component of load could potentially be addressed during childhood, through diet- or exercise-based weight loss programmes. It may be harder, and more controversial, to moderate high levels of LM and height, even though increased linear growth has been associated with adult BP in those born small [24]. We note than in industrialised populations, rapid infant growth has been associated with each of height, LM and FM [53], [54], [55], and some have proposed that slower growth is beneficial for reducing adult cardiovascular risk [5], [56]. However, in developing countries, infant weight gain and linear growth are beneficial for many outcomes [57], and it remains unclear whether infancy is an appropriate period for moderating metabolic load, hence childhood may be the optimal period for addressing metabolic load. A further caveat is that the associations of early growth and height in our sample may reflect effects on maturation rate as well as attained size, hence further follow-up is recommended to clarify the impact of infant and childhood growth on later metabolic load and BP.

In summary, our study used a conceptual model to help understand associations of early growth with later BP. Three different components of childhood metabolic load were each independently associated with BP and the odds of higher BP. The association between postnatal growth and BP could be attributed to these elements of metabolic load. Holding metabolic load constant, BP and the risk of higher BP were elevated in those with lower birth weight, representing reduced metabolic capacity.

## Supporting Information

Table S1 **Linear regression models for the prediction of systolic and diastolic blood pressure z-score and conditional pulse pressure by height, weight, lean and fat mass, birth length and conditional height velocity residual.**
(DOC)

Table S2 **Logistic regression for the odds of higher blood pressure.**
(DOC)
