# Supplementary material for: Testing a Capacity-Load Model for Hypertension: Disentangling Early and Late Growth Effects on Childhood Blood Pressure in a Prospective Birth Cohort
Source: PLoS One. 2013 Feb 6;8(2):e56078. doi: 10.1371/journal.pone.0056078 (PMC3566037; doi:10.1371/journal.pone.0056078)
Supplement: Table S1 — Linear regression models for the prediction of systolic and diastolic blood pressure z-score and conditional pulse pressure by height, weight, lean and fat mass, birth length and conditional height velocity residual. (DOC) [file pone.0056078.s001.doc]

**Table S1.** Linear regression models for the prediction of systolic and diastolic blood pressure z-score and conditional pulse pressure by height, weight, lean and fat mass, birth length and conditional height velocity residual.

|  |  | | | | | | | | | | | |
| --- | --- | --- | --- | --- | --- | --- | --- | --- | --- | --- | --- | --- |
|  | **DISTOLIC BLOOD PRESSURE (z-score)** | | | | | | | | | | | |
|  | *Males (n = 2,304)* | | | | | | *Females (n = 2,295)* | | | | | |
|  | Model 1 | | Model 2 | | Model 3 | | Model 1 | | Model 2 | | Model 3 | |
|  | β | 95% CI | β | 95% CI | β | 95% CI | β | 95% CI | β | 95% CI | β | 95% CI |
| Height* | 0.11 | ( 0.07; 0.14) |  |  | 0.16 | ( 0.04; 0.27) | 0.13 | ( 0.10; 0.16) |  |  | 0.22 | ( 0.12; 0.32) |
| LMr/H | 0.01 | (-0.02; 0.04) |  |  | 0.01 | (-0.02; 0.05) | 0.04 | ( 0.01; 0.07) |  |  | 0.03 | ( 0.00; 0.06) |
| FMr/LM | 0.14 | ( 0.11; 0.17) |  |  | 0.14 | ( 0.11; 0.17) | 0.15 | ( 0.12; 0.18) |  |  | 0.15 | ( 0.12; 0.18) |
| Birth length* |  |  | 0.01 | (-0.02; 0.04) | -0.04 | (-0.08; 0.01) |  |  | 0.01 | (-0.02; 0.03) | -0.04 | (-0.08; 0.00) |
| CHV |  |  | 0.10 | ( 0.07; 0.14) | -0.04 | (-0.15; 0.06) |  |  | 0.10 | ( 0.07; 0.13) | -0.08 | (-0.17; 0.01) |
| *r^2^* | 0.05 |  | 0.02 |  | 0.05 |  | 0.06 |  | 0.02 |  | 0.06 |  |
|  | **SYSTOLIC BLOOD PRESSURE (z-score)** | | | | | | | | | | | |
|  | *Males (n = 2,304)* | | | | | | *Females (n = 2,295)* | | | | | |
|  | Model 1 | | Model 2 | | Model 3 | | Model 1 | | Model 2 | | Model 3 | |
|  | β | 95% CI | β | 95% CI | β | 95% CI | β | 95% CI | β | 95% CI | β | 95% CI |
| Height* | 0.29 | ( 0.25; 0.33) |  |  | 0.36 | ( 0.22; 0.49) | 0.35 | ( 0.31; 0.38) |  |  | 0.49 | ( 0.37; 0.61) |
| LMr/H | 0.20 | ( 0.16; 0.24) |  |  | 0.20 | ( 0.17; 0.24) | 0.23 | ( 0.19; 0.27) |  |  | 0.22 | ( 0.18; 0.26) |
| FMr/LM | 0.27 | ( 0.23; 0.31) |  |  | 0.27 | ( 0.23; 0.30) | 0.29 | ( 0.25; 0.33) |  |  | 0.29 | ( 0.25; 0.32) |
| Birth length* |  |  | 0.04 | ( 0.00; 0.07) | -0.08 | (-0.13; -0.02) |  |  | 0.03 | (-0.01; 0.06) | -0.08 | (-0.13; -0.03) |
| CHV |  |  | 0.29 | ( 0.25; 0.33) | -0.04 | (-0.17; 0.09) |  |  | 0.28 | ( 0.24; 0.32) | -0.13 | (-0.24; -0.02) |
| *r^2^* | 0.19 |  | 0.08 |  | 0.19 |  | 0.22 |  | 0.07 |  | 0.22 |  |
|  | **PULSE PRESSURE (z-score)** | | | | | | | | | | | |
|  | *Males (n = 2,304)* | | | | | | *Females (n = 2,295)* | | | | | |
|  | Model 1 | | Model 2 | | Model 3 | | Model 1 | | Model 2 | | Model 3 | |
|  | β | 95% CI | β | 95% CI | β | 95% CI | β | 95% CI | β | 95% CI | β | 95% CI |
| Height* | 0.19 | ( 0.16; 0.23) |  |  | 0.21 | ( 0.09; 0.33) | 0.24 | ( 0.21; 0.27) |  |  | 0.30 | ( 0.19; 0.40) |
| LMr/H | 0.19 | ( 0.16; 0.22) |  |  | 0.19 | ( 0.16; 0.22) | 0.20 | ( 0.17; 0.24) |  |  | 0.20 | ( 0.16; 0.23) |
| FMr/LM | 0.14 | ( 0.11; 0.18) |  |  | 0.14 | ( 0.11; 0.17) | 0.17 | ( 0.13; 0.20) |  |  | 0.16 | ( 0.13; 0.20) |
| Birth length* |  |  | 0.03 | ( 0.00; 0.06) | -0.04 | (-0.09; 0.01) |  |  | 0.02 | (-0.01; 0.05) | -0.04 | (-0.08; 0.00) |
| CHV |  |  | 0.19 | ( 0.15; 0.22) | 0.00 | (-0.11; 0.11) |  |  | 0.20 | ( 0.16; 0.23) | -0.05 | (-0.15; 0.05) |
| *r^2^* | 0.12 |  | 0.05 |  | 0.12 |  | 0.15 |  | 0.05 |  | 0.15 |  |

β: mean difference in outcome per 1 unit exposure, 95% CI: 95% confidence intervals, LMr/H: Lean mass standardised residuals modelled on height; FMr/LM: Fat mass standardised residuals modelled on lean mass; CWV: Conditional height velocity residual.

* Birth length and height are expressed in z-scores
