# Supplementary material for: Testing a Capacity-Load Model for Hypertension: Disentangling Early and Late Growth Effects on Childhood Blood Pressure in a Prospective Birth Cohort
Source: PLoS One. 2013 Feb 6;8(2):e56078. doi: 10.1371/journal.pone.0056078 (PMC3566037; doi:10.1371/journal.pone.0056078)
Supplement: Table S2 — Logistic regression for the odds of higher blood pressure. (DOC) [file pone.0056078.s002.doc]

***Table S2.*** *Logistic regression for the odds of higher blood pressure.*

|  | | | | | | |
| --- | --- | --- | --- | --- | --- | --- |
| **DIASTOLIC BLOOD PRESSURE (z-score).** *Values ≥ 95^th^ centile = 108* | | | | | | |
|  | Model 1 | | Model 2 | | Model 3 | |
|  | OR | 95% C.I. | OR | 95% C.I. | OR | 95% C.I. |
| Height* | 1.21 | (0.99; 1.47) |  |  | 1.58 | (0.85; 2.94) |
| LMr/H | 1.10 | (0.92; 1.33) |  |  | 1.10 | (0.91; 1.32) |
| FMr/LM | 1.53 | (1.30; 1.80) |  |  | 1.51 | (1.28; 1.78) |
| Birth length* |  |  | 0.90 | (0.75; 1.08) | 0.80 | (0.62; 1.04) |
| CHV |  |  | 1.26 | (1.04; 1.53) | 0.81 | (0.46; 1.43) |
| Male sex | 1.04 | (0.71; 1.53) | 1.08 | (0.73; 1.58) | 1.02 | (0.69; 1.51) |
|  | | | | | | |
| **SYSTOLIC BLOOD PRESSURE (z-score).** *Values ≥ 95^th^ centile = 74* | | | | | | |
|  | Model 1 | | Model 2 | | Model 3 | |
|  | OR | 95% C.I. | OR | 95% C.I. | OR | 95% C.I. |
| Height* | 1.69 | (1.33; 2.15) |  |  | 2.33 | (1.12; 4.82) |
| LMr/H | 1.66 | (1.35; 2.05) |  |  | 1.66 | (1.34; 2.04) |
| FMr/LM | 1.80 | (1.50; 2.15) |  |  | 1.77 | (1.48; 2.12) |
| Birth length* |  |  | 0.93 | (0.75; 1.16) | 0.74 | (0.54; 1.00) |
| CHV |  |  | 1.81 | (1.44; 2.27) | 0.80 | (0.41; 1.56) |
| Male sex | 1.10 | (0.68; 1.76) | 1.19 | (0.75; 1.89) | 1.11 | (0.69; 1.79) |

OR: Odds ratio, 95% CI: 95% confidence intervals, LMr/H: Lean mass standardised residuals modelled on height; FMr/LM: Fat mass standardised residuals modelled on lean mass; CHV: Conditional height velocity. Higher blood pressure (BP) for each blood pressure component was defined using the cut-off criteria of ≥95^th^ centile as recommended by the Working Group on High Blood Pressure in Children and Adolescents. However, clinical diagnosis of hypertension uses a different measurement protocol to our epidemiological approach, hence although derived using the same cut-off values, our higher BP sample does not represent a clinical hypertension sample. n = 4,599.

* Birth length and height are expressed in z-scores
